# Identification of the Distinct Immune Microenvironment Features Associated with Progression Following High-Dose Melphalan and Autologous Stem Cell Transplant in Multiple Myeloma

**DOI:** 10.1158/2326-6066.CIR-25-0019

**Published:** 2025-05-08

**Authors:** Parvathi Sudha, Travis S. Johnson, Habib Hamidi, Ke Yang, Enze Liu, Brent Smith, Vivek Chopra, Michael Nixon, Faiza Zafar, Sherif S. Farag, Gareth J. Morgan, Ola Landgren, Kelvin Lee, Attaya Suvannasankha, Magdalena Czader, Rafat Abonour, Mohammad Abu Zaid, Brian A. Walker

**Affiliations:** 1 Division of Hematology and Oncology, Melvin and Bren Simon Comprehensive Cancer Center, School of Medicine, Indiana University, Indianapolis, Indiana.; 2 Department of Biostatistics and Health Data Sciences, School of Medicine, Indiana University, Indianapolis, Indiana.; 3 Genentech Inc., South San Francisco, California.; 4 Department of Pathology and Laboratory Medicine, Indiana University School of Medicine, Indianapolis, Indiana.; 5 Myeloma Research Program, NYU Langone, Perlmutter Cancer Center, New York, New York.; 6 Division of Myeloma, Sylvester Comprehensive Cancer Center, University of Miami, Miami, Florida.; 7 Center for Computational Biology and Bioinformatics, School of Medicine, Indiana University, Indianapolis, Indiana.

## Abstract

A key treatment for patients with multiple myeloma is high-dose melphalan followed by autologous stem cell transplant (ASCT). It can provide a deep response with long-term remission. However, some patients progress quickly, and it is not clear why. In this study, we performed single-cell RNA and T-cell receptor sequencing of the immune microenvironment of 40 patients before and after ASCT to determine if differences in the immune composition could define those who would progress. Clear differences in cell populations were identified in progressors, including increased T-cell infiltration, decreased T-cell receptor diversity, and decreased frequency of monocytes and CD56^bright^ NK cells. We identified cell interactions that predicted progression, including increased frequency of CD8^+^ exhausted T cells and stromal cells and decreased frequency of CD56^bright^ NK cells and plasmacytoid dendritic cells. We propose and validate a model of progression that can also be determined by flow cytometry. Together, these data highlight the importance of the immune microenvironment in understanding responses to ASCT.

## Teaser

The authors investigate why some patients with multiple myeloma rapidly progress after autologous stem cell transplant, finding they have unique features in the reconstituted immune microenvironment, including increased T-cell subsets, decreased monocytes, and decreased NK-cell populations.

## Introduction

Multiple myeloma is a hematologic cancer characterized by malignant plasma cell accumulation in the bone marrow (1). Following induction treatment, high-dose melphalan and autologous stem cell transplant (ASCT) remain cornerstones of therapy for eligible newly diagnosed patients with multiple myeloma (2); however, patients ultimately progress. Clearly, there are tumor intrinsic and extrinsic factors that drive progression, including high-risk genomics of the tumor and factors in the bone marrow microenvironment.

The interaction of normal plasma cells with their supportive microenvironment is crucial for plasma cell longevity (3). A characteristic feature of myeloma cells is the requirement for an intimate relationship with the bone marrow microenvironment, in which plasma cells are nurtured in specialized niches that facilitate growth, maintain survival, and aid in resistance to treatment of the myeloma clone (4). The microenvironment includes endothelial cells and adipocytes, as well as the immune microenvironment that can be suppressed, fostering suitable conditions for tumor growth (5). An imbalance in these interactions is important in the immortalization of a myeloma-propagating cell. Different cell types in the microenvironment have distinct roles in controlling tumor cells, and by understanding these relationships, we may be able to design clinical trials in which the aim is to improve immune control and eradication of tumor cells. These relationships cannot be fully evaluated using bulk sequencing techniques, as these convolute the signal from all immune cell types. By using single-cell sequencing technologies on patient bone marrow samples before and after ASCT, we can comprehensively characterize the different components of the immune microenvironment affecting progression after ASCT.

We performed an extensive study to investigate the role of the microenvironment in response to high-dose melphalan using single-cell RNA (scRNA) and T-cell receptor (TCR) sequencing in paired pre- and post-ASCT bone marrow samples. Using this comprehensive dataset, we evaluated TCR clonal expansions and cell type proportional changes between those who progressed and those who did not. Analysis of both pre- and post-ASCT samples essentially created duplicate data to strengthen our results. From these analyses, we identified robust shifts in the microenvironment that may influence the risk of progression following ASCT.

## Materials and Methods

### Patient samples

This study included 40 patients with multiple myeloma (39 newly diagnosed and one relapse) who had undergone stem cell transplant and were enrolled between October 2018 and July 2021 in the Indiana Myeloma Registry, a prospective noninterventional study approved by the Institutional Review Board of Indiana University, collecting comprehensive clinical, genomic, demographic, social, environmental, and quality of life data from subjects with plasma cell dyscrasias. All patients gave informed consent for sample use and data collection. This study was performed in accordance with the Declaration of Helsinki. All patients received pre-ASCT induction regimens that included proteasome inhibitors, immunomodulatory drugs (IMiD), and steroids, with 36 of the 40 patients having received an IMiD as part of their induction therapy. Bone marrow aspirates were collected from 40 patients before (range 4–181 days prior to transplant) and after high-dose melphalan and ASCT (range 58–142 days after transplant; Supplementary Fig. S1A; Supplementary Table S1A–S1D). Five patients progressed quickly, and the post-ASCT sample time point was also the progression sample, so these five samples were included in progressor versus nonprogressor cluster comparisons but not in model generation.

### Cell preparation

Bone marrow aspirates were collected in heparin tubes from patients with multiple myeloma and were isolated by Ficoll-Paque PLUS (General Electric). Briefly, bone marrow aspirates were diluted in phosphate-buffered saline, layered over Ficoll-Paque PLUS, and centrifuged at 400 × *g* for 30 minutes at room temperature. Mononuclear cells were collected and washed. Red blood cells were removed by the addition of red blood cell lysis buffer (Roche), followed by centrifugation and washing. Cells underwent CD138^+^ selection (CD138 MicroBeads, Miltenyi Biotec), and positive and negative fractions were viably frozen until processed for library construction (6).

### Sequencing library construction

From the CD138^−^ fraction of the bone marrow cells, single-cell cDNA synthesis, amplification, and sequencing libraries were generated using either the Single Cell 5′ Reagent or 3′ Reagent Kit (10x Genomics) following the manufacturer’s instructions. Briefly, cryopreserved cells were thawed and processed as previously described (6). The final single-cell suspension was assessed for viability, and if <70% of viable cells are present, a dead cell depletion procedure was applied (Miltenyi Biotec). A total of 10,000 cells were processed for gel bead-in emulsion generation and barcoding, followed by cDNA synthesis and amplification, and ∼5% of the resulting cDNA was amplified using the TCR probe set for the TCR-specific library (Chromium Single Cell Human TCR Amplification Kit, 10x Genomics), whereas the remaining cDNA was used for 5′ library preparation (Chromium Next GEM Single Cell 5′ kit, 10x Genomics). At each step, the quality of the cDNA and that of the library were examined by Bioanalyzer and Qubit. For each sample, each resulting TCR and 5′ library was pooled in a ratio of 15% and 85%, respectively. The scRNA libraries were pooled and sequenced on an Illumina HiSeq 3000 system using 26 and 90 bp paired-end reads, giving ∼50,000 reads per cell.

### Single-cell data analysis

Samples were aligned using the Cell Ranger multi pipeline (v6.1.2), with VDJ libraries listed as “vdj-t” to indicate T-cell libraries. The reference transcriptome was generated using the Cell Ranger “makeref” command for genome assembly hg38 and Ensembl v94 as the reference set of annotations. After alignment, the filtered_feature_bc_matrix.h5 files from Cell Ranger multi pipeline were loaded into Seurat. Cells with <500 genes detected were filtered to remove low-quality cells, and cells with aberrantly high gene counts were also removed to filter out doublets. Cells with more than 10% of reads mapped to mitochondrial genes were removed because they were more likely to be necrotic or of poor quality. Samples were independently normalized using the SCTransform function in Seurat v4 (7). Integration of all 80 samples was performed using the top 3,000 variable genes from each dataset. Using k-nearest neighbors (kNN) and shared-nearest neighbors graphs (FindIntegrationAnchors function), anchors (pairwise cell correspondence between datasets) were found and used to transform the data into an integrated space. Principal component analysis was performed on these integrated data, and kNN with a resolution of 0.8 was used to identify clusters. CD138^+^ and red blood cell clusters were removed from the analysis before performing a second round of data processing using Seurat and reclustering cells based on expression profiles. Cell types were annotated using marker genes from the Azimuth human bone marrow database (8). The “FindMarkers” function was used to conduct differential gene expression analysis between each group of annotated clusters of interest.

Cell cluster–specific marker genes were identified by running the “FindAllMarkers” function from the Seurat package on the normalized gene expression data. Differentially expressed genes between two clusters were identified using the “FindMarkers” function. Biological process enrichment analysis was performed using gene set enrichment analysis.

The proportion of cells in each cell type was calculated and visualized using dittoSeq (9). The mean proportion of cells per sample was compared for each response group, and significant differences were visualized by violin plots. T-cell analysis was performed by extracting the T lineage cells from the integrated dataset and clustering them separately using kNN clustering. TCR data were analyzed using the full dataset with scRepertoire (v1.6; ref. 10) and immunarch (v1.0.0; ref. 11). Based on the frequency of the clonotype occurrences, they were annotated as hyperexpanded (100 < X ≤ 500), large (20 < X ≤ 100), medium (5 < X ≤ 20), small (1 < X ≤ 5), and single (X = 1). Repertoire basic statistics and diversity calculations were performed using immunarch with the “repDiversity” function. A nonparametric asymptotic estimator of species richness, “chao1”, was used for clonotype diversity calculations.

### Next-generation flow for measurable residual disease analysis

Bone marrow samples were collected in EDTA tubes after ASCT for 35 patients without progressive disease. Cells were clinically assessed for measurable residual disease using the two-tube EuroFlow immunophenotyping methodology (12) on Gallios and Navios EX flow cytometers (Beckman Coulter) using the multiple myeloma measurable residual disease (MRD) kit (BD Cytognos) containing antibodies specific for CD38, CD56, CD45, CD19, CD117, CD81, cytoKappa and cytoLambda with add-on antibodies specific for CD138 (BV421, BD Horizon), and CD27 (BV510, BioLegend; Supplementary Fig. S2). In addition to reporting MRD status, flow data were reanalyzed for percentages of mature B cells, B-cell progenitors, T/NK cells, myeloid precursors, monocytes, and both normal and abnormal plasma cells. Granulocytes were removed from the analysis for comparison with single-cell data in which samples had undergone Ficoll separation.

### Statistical tests between groups

Statistical significance between each group of annotated clusters was calculated by determining the proportion of each cluster per patient and comparing the medians of the two groups [progressors (*n* = 19) vs. nonprogressors (*n* = 21)] using an unpaired *t* test, and *P* values < 0.05 were considered statistically significant. The FDR was also calculated for these comparisons. We considered a comparison significant if *P* ≤ 0.05 and FDR ≤0.25. For categorical data, the Fisher exact test was used.

For the optimal forest plot, outcome-oriented methods were applied to each cell type to determine a threshold that corresponded to the most significant relation with progression-free survival (PFS) for each variable. HRs were calculated based on Cox regression of PFS, comparing patients above the threshold with those below for each gene cell type.

### Survival analysis

Survival analysis was performed using the most common nonparametric approach, the Kaplan–Meier method. Progression status and time to progression were used. Survival analysis was conducted using R with the “survival” (v3.5.8) package.

### LASSO Cox regression analysis

The proportions of all cell types, the CD56^bright^/CD56^dim^ ratio, TCR diversity, and age from each sample, along with disease progression time, were used to create Least Absolute Shrinkage and Selection Operator (LASSO) Cox regression models to study cellular interactions. Specifically, all cell types and all cell type pairwise interactions were included in the LASSO Cox models. Patients who had progressed at the post-ASCT time point (*n* = 5) were removed from the prediction model. Leave-one-out cross-validation was used to generate multiple LASSO Cox models, and the number of positive and negative occurrences of each coefficient was counted to identify potential cellular interactions that may be correlated with progression.

To validate our model of prognosis, the cell type proportions from a previously published dataset (13) were used from the posttreatment samples of patients who received daratumumab, carfilzomib, lenalidomide, and dexamethasone, with corresponding PFS, response, and MRD information. First, of all the models trained during leave-one-out cross-validation, we selected the model with the highest R^2^ value from our training cohort. Cell types were harmonized to create comparable feature sets to test our model against the dataset of Maura and colleagues (Supplementary Table S2). Finally, the model coefficients from our highest R^2^ model were used, without any additional model training, to predict a hazard value for each of the posttreatment patients. As there were fewer progression events in the validation dataset, we used a more stringent high-risk cutoff of the 80th percentile of hazard scores such that the patients in the top 20% of hazard scores were considered high-risk. Log-rank tests were used to evaluate patient stratification. Besides the survival analysis with high sparsity, we also evaluated whether the hazard values were higher for patients with partial response (PR) versus complete response and for patients with sustained MRD negativity versus unsustained MRD negativity. Nonparametric Wilcoxon rank sum tests were used to evaluate differences in hazard between groups of patients.

### Cell–cell interaction analysis

Cell–cell interaction analysis for the immune cells was performed using CellChat (v2; ref. 14). Cells from progressor and nonprogressor samples after ASCT underwent ligand–receptor analysis. Using CellChat’s standard workflow, aggregated cell–cell communication networks, the number of interactions and interaction strength, the contribution of ligand–receptor pairs to the overall signaling, gene expression distribution in each cell type, and signaling roles (dominant senders and receivers) of cell types were analyzed. Signaling pathways with larger differences between groups were identified by computing the distance of overlapped signaling pathways.

### Data availability

Microenvironment single-cell genomic data are available at the Database of Genotypes and Phenotypes with accession number phs003219.v1.p1. Data can be accessed by submitting a request to the Data Access Committee at the Database of Genotypes and Phenotypes. Data from the validation dataset were previously published and can be accessed through the European Genome-phenome Archive with accession number EGAD00001011132. All other data are available in the article and its supplementary files or from the corresponding author upon reasonable request.

## Results

### Immune cell type composition differs between progression states

**Figure 1.**
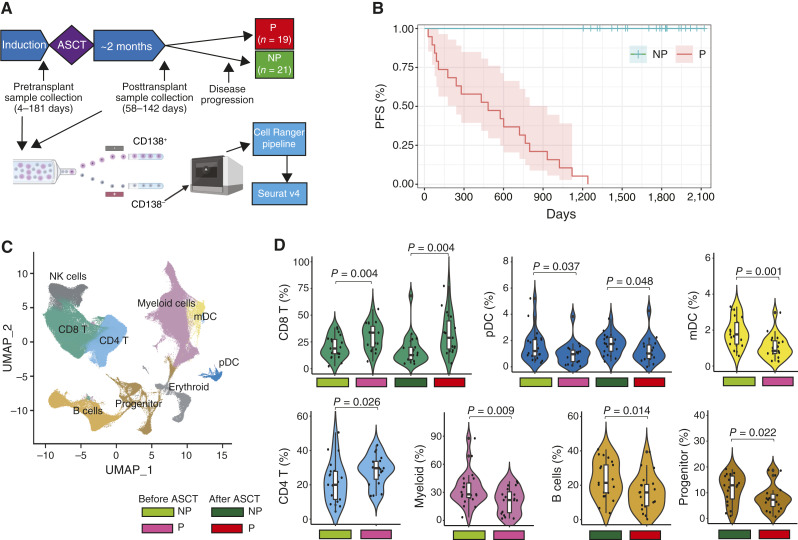
Single-cell transcriptomic profiling of the bone marrow microenvironment before and after ASCT identifies cell types associated with progression. **A,** Overview of the study. **B,** PFS Kaplan–Meier plot for study patients. **C,** Patient-integrated annotated cell clusters. **D,** Mean proportions of cell types in progressor (P) and nonprogressor (NP) patients.

Transcriptomic profiling of CD138^–^ bone marrow was performed using 80 samples from 40 patients before or after ASCT (Fig. 1A), giving 485,316 cells for analysis that passed stringent quality control. Patients were followed and grouped into those who progressed (*n* = 19) and those who did not progress (*n* = 21) according to International Myeloma Working Group criteria (15). For progressors, the median time to progression was 483 days (range 28–1,240 days), and for nonprogressors, the median time to progression was not reached, and the median follow-up duration was 1,786 days (range 1,205–2,123 days; Fig. 1B). Patient demographic information is shown in Table 1, with no significant differences in gender (*P* = 0.9), age >65 years (*P* = 0.4), race (*P* = 0.4), IMiD maintenance (*P* = 0.12), or International Staging System (*P* = 0.3) between the two groups.

**Table 1.** Patient demographics.

	Total (*n* = 40)	Progressor (*n* = 19)	Nonprogressor (*n* = 21)	*P value*
Male (*n*, %)	26 (65%)	13 (68.4%)	13 (61.9%)	0.921
Age >65 y (*n*, %)	12 (30%)	9 (47.3%)	3 (14.3%)	0.359
International Staging System (ISS)				0.3053
ISS I (*n*, %)	10 (25%)	4 (21%)	6 (28.6%)	
ISS II (*n*, %)	11 (27.5%)	4 (21%)	7 (33.3%)	
ISS III (*n*, %)	7 (17.5%)	5 (26.3%)	2 (9.5%)	
Not available	12 (30%)	6 (31.6%)	6 (28.6%)	
Race				0.402
White (*n*, %)	35 (87.5%)	18 (94.7%)	17 (81%)	
Black (*n*, %)	5 (12.5%)	1 (5.3%)	4 (19%)	
IMiD maintenance	22 (55%)	8 (42.1%)	14 (66.6%)	0.12

Two-dimensional embedding using Uniform Manifold Approximation and Projection for Dimension Reduction (UMAP) based on single-cell gene expression identified bone marrow immune cell types (Fig. 1C), including CD4^+^ T cells, CD8^+^ T cells, NK cells, B cells, monocytes/myeloid cells, myeloid dendritic cells (mDC), plasmacytoid dendritic cells (pDC), and their progenitor populations.

There were many differences in cell type proportions between the pre- and post-ASCT samples, including a significant increase in B cells (2.6% vs. 19.6%; *P* < 0.001) and all progenitor cells (1.5% vs. 8.9%; *P* < 0.001) and a significant decrease in CD4^+^ T cells (22.5% vs. 9.5%; *P* < 0.001) and monocyte/myeloid cell populations (27.8% vs. 21%; *P* = 0.007) after ASCT (Supplementary Fig. S1).

To ascertain if the tumor microenvironment is associated with disease progression, we determined the proportion of each cell type in progressor and nonprogressor samples at both pre- and post-ASCT time points (Fig. 1D). In the pre-ASCT samples, significantly different proportions of CD4^+^ T cells, CD8^+^ T cells, mDCs, pDCs, and myeloid cells were identified (*P* < 0.01), and in the post-ASCT samples, significant differences in B cells, CD8^+^ T cells, pDCs, and progenitor cells were identified (Supplementary Table S3). The distribution of proportions in each sample is shown in Fig. 1D. Of note, CD8^+^ T cells and pDCs were significantly different between progressors and nonprogressors at both the pre- and post-ASCT time points, showing concordance of data. The CD8^+^ T-cell compartment was significantly lower in nonprogressors at both time points (before ASCT: median 33.6% vs. 18.8%; *P* = 0.004; after ASCT: 28.8% vs. 12.6%; *P* = 0.004), whereas pDCs were significantly higher in nonprogressors (before ASCT: 0.99% vs. 1.20%; *P* = 0.037; after ASCT: 1.01% vs. 1.74%; *P* = 0.048). Most of the other significant differences were seen in the pre-ASCT samples, with CD4^+^ T cells (29.7% vs. 20.0%; *P* = 0.026) and mDCs (0.85% vs. 1.76%; *P* = 0.001) being lower in nonprogressors and myeloid cells (21.7% vs. 27.88%; *P* = 0.009) being increased in nonprogressors. In post-ASCT samples, B cells (15.79% vs. 21.39%; *P* = 0.014) and progenitor cells (7.26% vs. 12.87%; *P* = 0.022) were significantly increased in nonprogressors.

### Next-generation flow cytometry posttransplant can predict the chance of progression

**Figure 2.**
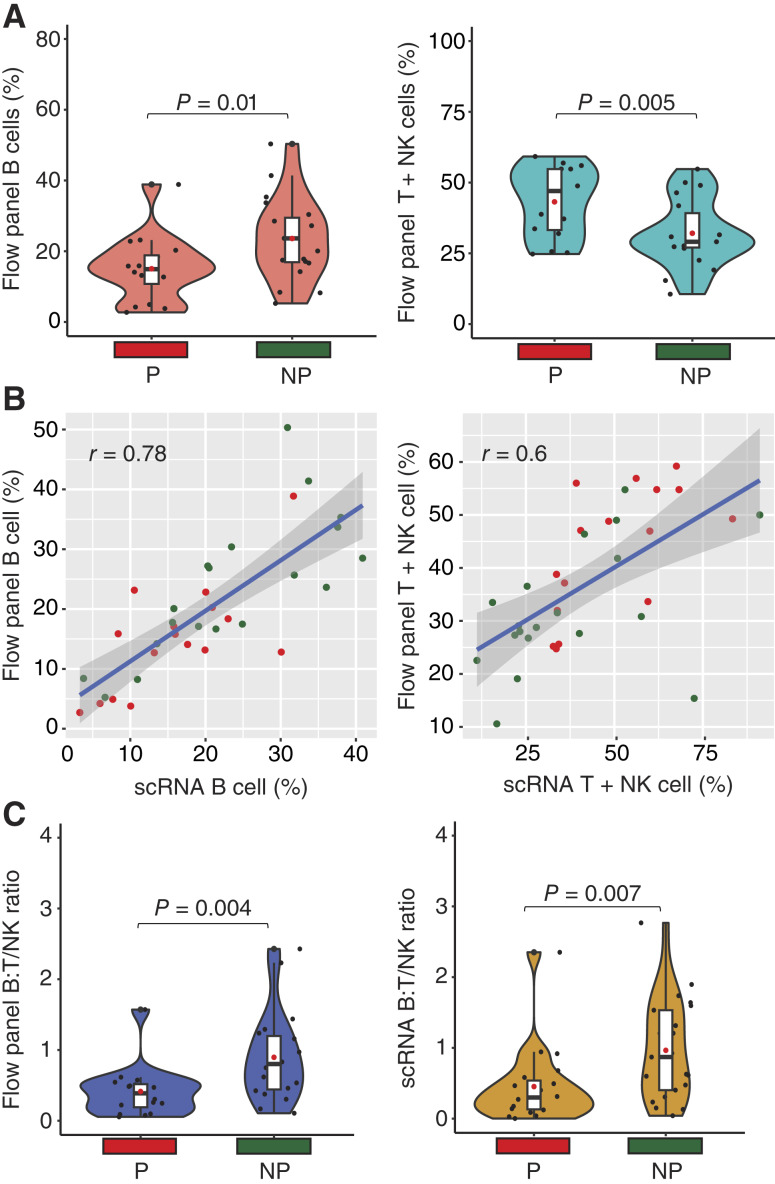
B cell to T cell/NK cell ratio (B:T/NK) is associated with progression after ASCT. **A,** MRD flow panel percentages of B and T+NK cells after ASCT. **B,** Correlation of flow panel and single-cell percentages. **C,** The ratio of B/T+NK cells is associated with progression in both flow panel and single-cell datasets. NP, nonprogressor, P, progressor.

We leveraged the clinical MRD next-generation flow cytometry performed by our pathology department to validate the changes in cell types seen in the scRNA sequencing data. Granulocytes were removed from the flow panel data to match the single-cell samples, which had undergone Ficoll separation. When progressor and nonprogressor samples were compared using flow panel data, the nonprogressor samples had significantly more B cells (23.6% vs. 14.9%; *P* = 0.01) and fewer T/NK cells (29.1% vs. 47.0%; *P* = 0.005; Fig. 2A). There was no difference in time to sampling after ASCT between progressors and nonprogressors that may account for the difference in cell proportions (Supplementary Fig. S1). The proportions of B and T/NK cell subsets also correlated well between the flow panel and single-cell datasets (B cell: *r* = 0.78; T/NK cell: *r* = 0.60; Fig. 2B). Given that the proportion of B and T/NK cells was likely inversely related, we calculated the ratio of B:T/NK cells in both the flow and single-cell datasets and found that nonprogressors had a higher ratio compared with progressors (flow panel: 0.80 vs. 0.38; *P* = 0.004; single-cell data: 0.87 vs. 0.29; *P* = 0.007; Fig. 2C). The decreased ratio of B:T/NK cells as a marker of progression was only seen in the post-ASCT samples and not in the pre-ASCT samples, so it could not be used as a predictor of progression before ASCT.

### Defining subgroups of cell types associated with disease progression

**Figure 3.**
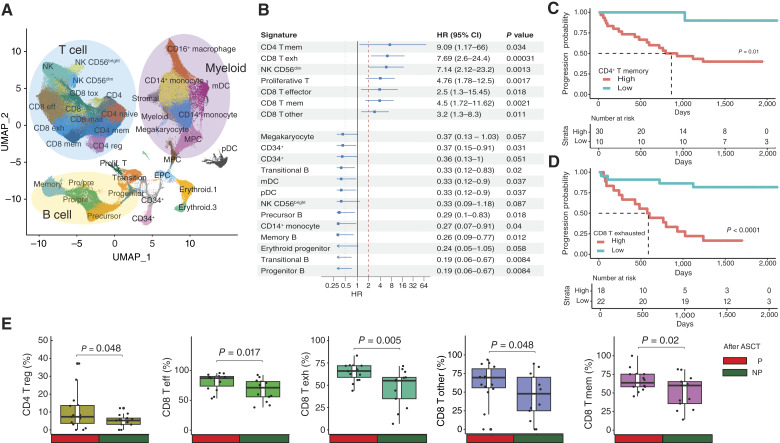
Time to progression correlates with decreased T-cell frequencies and increased monocyte frequencies. **A,** Identification and mapping of cell-type subclusters. **B,** Optimal forest plot of cell types associated with outcomes in univariate analysis. **C,** High CD4^+^ T memory cells are associated with early progression after ASCT. **D,** High CD8^+^ exhausted T cells are associated with early progression after ASCT. **E,** Significant differences in expanded clonotype T-cell proportions between nonprogressors (NP) and progressors (P). CI, confidence interval; T eff, effector T cell; T exh, exhausted T cell; T mem, memory T cell; T reg, regulatory T cell.

To investigate further the B- and T-cell populations associated with progression, we identified subpopulations of cell types based on their transcriptomic profiles. We identified 35 clusters, which included subpopulations of B cells, CD4^+^ and CD8^+^ T cells, myeloid cells, NK cells, and progenitor cells (Fig. 3A). Using transcriptomic data, the cell subpopulations were identified (Supplementary Figs. S3 and S4), and the proportion of cells clustered in pre- and post-ASCT samples was compared based on their progression status (Supplementary Fig. S3; Supplementary Table S4).

We observed that six subclusters were significantly changed in proportion between progressors and nonprogressors in both the pre- and post-ASCT samples (*P* < 0.05, FDR < 0.25), indicating a consistent relationship with disease progression (Supplementary Table S4). These clusters were exhausted CD8^+^ T cells (before ASCT: 8.29% vs. 6.05%; *P* = 0.021; after ASCT: 7.55% vs. 4.24%; *P* = 0.001) and memory CD8^+^ T cells (before ASCT: 3.46% vs. 1.63%; *P* = 0.009; after ASCT: 2.28% vs. 1.33%; *P* = 0.025), which were all lower in nonprogressors, as well as pDCs (before ASCT: 0.98% vs. 1.59%; *P* = 0.037; after ASCT: 1.24% vs. 1.71%; *P* = 0.048), CD14^+^ monocytes (before ASCT: 4.86% vs. 9.89%; *P* = 0.005; after ASCT: 4.56% vs. 6.46%; *P* = 0.038), myeloid progenitors (before ASCT: 2.24% vs. 4.01%; *P* = 0.019; after ASCT: 2.64% vs. 3.89%; *P* = 0.022), and B-cell progenitors (before ASCT: 0.04% vs. 0.12%; *P* = 0.016; after ASCT: 0.72% vs. 1.33%; *P* = 0.005), which were all increased in nonprogressors. Taken together, these data indicate a link between progression and more exhausted and memory CD8^+^ T cells and fewer B-cell progenitors, myeloid progenitors, and monocytes in the bone marrow microenvironment.

As the flow panel data indicated an association with progression in the post-ASCT samples, we looked to see what else was significantly changed (*P* < 0.05, FDR < 0.25) between progressors and nonprogressors at this time point (Supplementary Table S4). In the post-ASCT samples, precursor B cells (6.11% vs. 10.28%; *P* = 0.013), myeloid CD14^+^ monocyte cluster cells (0.19% vs. 0.37%; *P* = 0.05), and pre–/pro–B cells (2.55% vs. 4.16%; *P* = 0.011) were higher in nonprogressors, whereas CD8^+^ T effector cells (14.43% vs. 7.29%; *P* = 0.006), NK CD56^dim^ cells (1.05% vs. 0.69%; *P* = 0.015), and proliferative T cells (0.31% vs. 0.18%; *P* = 0.027) were lower in nonprogressors.

Taken together, there was a tendency toward higher T-cell frequencies and lower myeloid-cell frequencies in nonprogressors compared with those who progressed. In addition, progenitor B and precursor B cells were found at lower frequencies in progressors at the post-ASCT time point.

### Early progression is associated with increased T-cell subsets

Next, we wanted to know if the cell-type proportions were associated with time to progression. We first determined the optimal cut point for cell types such that it corresponded with the most significant correlation with time to progression. Patients were binned into high or low categories based on the cutoff, and a Cox regression analysis was performed. Patients with high proportions in seven of the cell types (of which six were T-cell subsets) had a significantly higher risk of progression after ASCT (Fig. 3B) than those with low proportions. These T-cell subsets included CD4^+^ memory, CD8^+^ exhausted, CD8^+^ memory, and other CD8^+^ T cells. The two subsets most significantly associated with progression (highest HRs) were CD4^+^ memory T cells (median time to progression 865 days vs. not reached; *P* = 0.01) and CD8^+^ exhausted T cells (588 days vs. not reached; *P* < 0.0001; Fig. 3C and D).

### Increased TCR expansion is associated with progression

Using the TCR sequencing data, we examined the dataset for clonotype expansion (Supplementary Fig. S5). TCR sequences were analyzed, identifying a total of 72,005 unique rearrangements across all samples. These clonotypes were annotated to determine if they were single or expanded in each patient. The majority of expanded clonotypes were detected in CD8^+^ T cells (96% CD8 vs. 4% CD4 T cells). At each time point, there was no difference in the total number of unique clonotypes per sample in the progressor and nonprogressor patients.

We went on to examine the proportion of each T-cell subgroup that was expanded versus a single clonotype. In the pre-ASCT samples, there was no significant difference between those who progressed or did not progress and the proportion of expanded clonotypes. However, in the post-ASCT samples, several T-cell subsets showed increased proportions of expanded clonotypes in the progressors compared with the nonprogressors (Fig. 3E), including CD4^+^ regulatory T cells (7.3% vs. 5.2%; *P* = 0.048) and many CD8^+^ T-cell subsets, including CD8^+^ exhausted T cells (66.0% vs. 55.1%; *P* = 0.005), CD8^+^ memory T cells (63.6% vs. 60.0%; *P* = 0.02), CD8^+^ effector T cells (87.4% vs. 71.2%; *P* = 0.017), and other CD8^+^ T cells (69.6% vs. 47.8%; *P* = 0.048). This indicates that post-ASCT T-cell clonotypes are more likely to be expanded in those who progress, especially among exhausted T cells.

Once again, both the pre- and post-ASCT samples show an increase in T-cell subsets and a decrease in myeloid cells in progressors, and this change is accompanied by an increase in expanded T-cell clonotypes.

### NK cell subsets are associated with progression status

**Figure 4.**
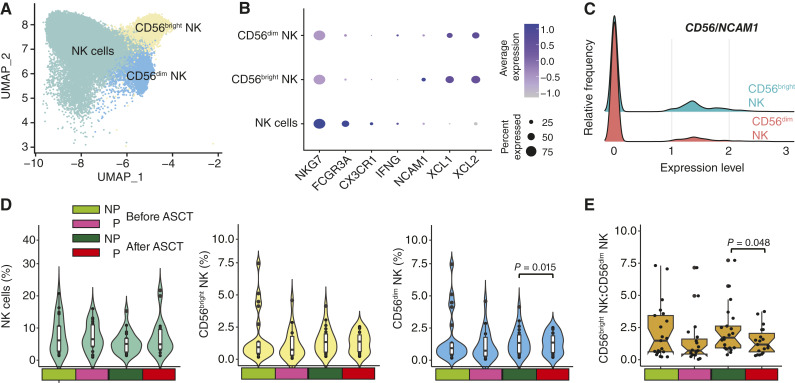
NK cell CD56^bright^/CD56^dim^ ratio is associated with progression. **A,** UMAP of NK cells identifying three populations. **B,** NK cell populations are defined by the expression of *CD56* (*NCAM1*) and *NKG7*. **C,** Expression of *CD56* (*NCAM1*) in *CD56*^bright^ and *CD56*^dim^ cells. **D,** NK cell populations do not differ between progression states except for *CD56*^dim^ NK cells after ASCT. **E,** The ratio of *CD56*^bright^ to *CD56*^dim^ cells is significantly different after ASCT. NP, nonprogressors; P, progressors.

It has previously been shown that NK cell subsets and ratios of *CD56*/*NCAM1* bright to dim cells were associated with response (13). Among the NK cells (Fig. 4A), three populations were detected based on key markers: *CD56*^bright^, *CD56*^dim^, and other NK cells (Fig. 4B and C). By examining the difference in means between progressors and nonprogressors, we did not see any differences in the NK or CD56^bright^ populations, but there was a significant decrease in *CD56*^dim^ NK cells at the post-ASCT stage in nonprogressors (0.826% vs. 0.604%; *P* = 0.015; Fig. 4D), which ties in with the increased T-cell and NK cell populations in progressors as seen by flow cytometry in Fig. 2. This change in CD56^dim^ NK cells also resulted in a significant difference in the ratio of *CD56*^bright^/*CD56*^dim^ cells (1.21 in those who progressed vs. 1.71 in those who did not progress; *P* = 0.048; Fig. 4E).

### Cell–cell interactions predict T-cell signaling pathways associated with disease progression

**Figure 5.**
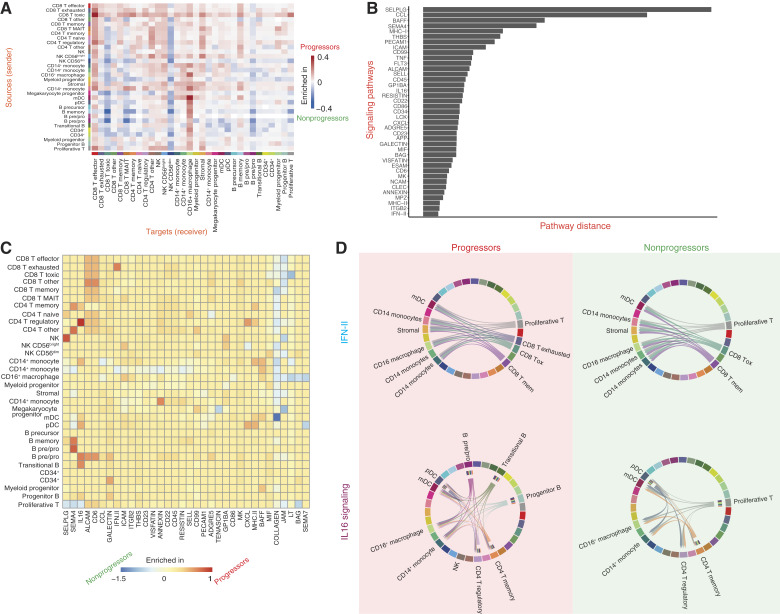
Cell–cell interaction networks of disease progressors and nonprogressors after ASCT. **A,** Differential heatmap comparing the interaction weights between each cell type in progressors and nonprogressors. Rows correspond to different sender cells (ligands), and columns correspond to receivers (receptors). **B,** Bar plot showing signaling networks with larger (or fewer) differences based on their Euclidean distance between progressors and nonprogressors. **C,** Heatmap with differential pathway enrichment between progressors and nonprogressors. **D,** IFN-γ and IL16 pathways show more cell–cell interactions in progressors compared with nonprogressors. mem, memory; tox, toxic.

We further investigated the cell–cell communications for similarities and differences in the immune microenvironment between the progressors and nonprogressors for the post-ASCT samples. Based on the ligand–receptor expression, an information flow score was computed for each ligand–receptor pathway between all cell types, and interaction patterns were identified using CellChat (14). Overall, the signaling structure was similar between progressors and nonprogressors (Fig. 5A), with some important differences, including higher sender signals for CD8^+^ T cytotoxic cells and CD14^+^ monocytes, whereas CD8^+^ T effector cells and CD16^+^ macrophages received higher signals in progressors.

In nonprogressors, B cells sent higher signals, whereas CD8^+^ T toxic cells, CD56^dim^ NK cells, and pre–/pro–B cells received higher signals (Fig. 5A). For the common pathways in both progressors and nonprogressors, the signaling networks with larger differences are seen in SELPLG, CCL, BAFF, and SEMA4 signaling pathways (Fig. 5B). IFN-II (IFN-γ), IL16, SEMA4, ALCAM, and CD6 pathways showed a higher interaction strength in disease progressors, whereas COLLAGEN, junctional adhesion molecule, lymphotoxin, SEMA7, and TENASCIN signaling pathways were only present in the nonprogressors (Fig. 5C).

IFN-γ and IL16 were identified as signaling networks with the highest difference between disease progressors and nonprogressors. IFN-γ signaling was increased in CD8^+^ exhausted T cells in progressors (Fig. 5C), and upon examination of the interactions, there was increased signaling among those cells and mDCs, stromal cells, CD14^+^ monocytes, and CD16^+^ macrophages (Fig. 5D). IFN-γ signaling is known to restrict antitumor responses by inhibiting the maintenance and diversity of intratumoral stem-like T cells (16). We also observed differential expression of IL16 in CD4^+^ T regulatory cells, pre–/pro–B cells, and progenitor cells, which was increased in progressors and is in line with previous studies (17).

### Determining a model of progression

**Figure 6.**
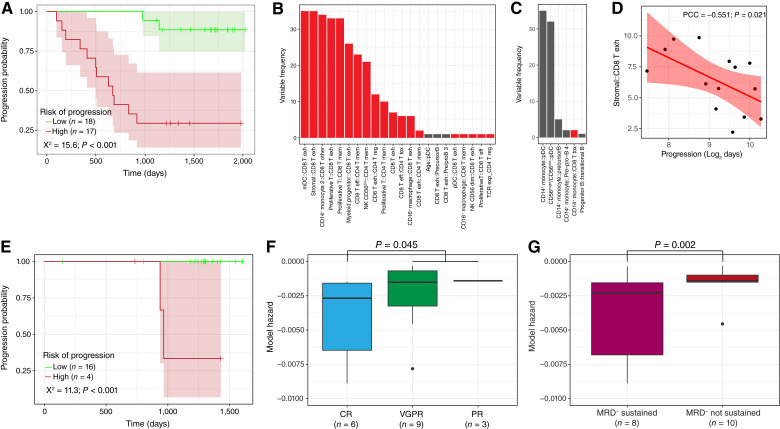
Modeling progressive disease based on immune cell composition. **A,** Kaplan–Meier curve of high risk (*n* = 17) and low risk (*n* = 18) of progression-defined groups. Shading represents 95% confidence intervals. **B,** Model prediction of cell interactions positively associated with progression. **C,** Model prediction of cell interactions negatively associated with progression. Those in red involve T-cell subsets. **D,** Correlation between the stromal::CD8 T exhausted cell proportions and progression. **E,** Kaplan–Meier curve of high and low risk of progression groups from the validation cohort. **F,** Proportion of model HR for complete response (CR; *n* = 8), very good PR (VGPR; *n* = 9), or PR (*n* = 3; *P* = 0.045). **G,** Proportion of model HR for patients with sustained MRD negativity (*n* = 10) and without sustained MRD negativity (*n* = 10; *P* = 0.002). The bottom and top edges of the box represent the 25th and 75th quartiles, respectively, and the middle line denotes median. The whiskers extend to 1.5 times the interquartile range of 25th and 75th quartiles. [Note: the plots do not include two outlier points (−0.113 and −0.031) from MRD-sustained and CR patients because their hazard scores were so low that it affected the plot visualization.] PCC. Pearson correlation coefficient; T eff, effector T cell; T exh, exhausted T cell; T mem, memory T cell; T reg, regulatory T cell; T tox, toxic T cell.

Using 35 of the 40 patients at the post-ASCT time point, after removing those who had already progressed, we defined a model of progression using the outcome and immune population data. After ASCT, we performed a leave-one-out cross-validation and were able to stratify the patients based on the predicted risk of progression (*P* < 0.001, *χ*^2^ = 15.6; Fig. 6A). Retained terms associated with increased risk were exhausted T cells in combination with other cell types as interaction terms (Fig. 6B). Notably, the exhausted T cell::stromal cell and exhausted T cell::mDC proportions were synergistic and occurred in 35/35 models with and without the main effect of exhausted T cell included (Fig. 6B). All but three terms associated with increased risk of progression involved increased T cells. There were also multiple terms associated with decreased risk, including pDC interactions with either CD14^+^ monocytes or the CD56^bright^/CD56^dim^ ratio of NK cells (Fig. 6C). Only one term associated with decreased risk of progression involved T cells. A correlation between the interaction of stromal cells and CD8^+^ exhausted T cells was seen with time to progression (Fig. 6D), again showing the importance of exhausted T cells and highlighting possible interactions with other cells in the microenvironment.

The progression model based on microenvironment cell types was validated using posttreatment samples (*n* = 20 with single-cell data) from an external dataset of patients who received daratumumab, carfilzomib, lenalidomide, and dexamethasone (after eight cycles; ref. 13). Based on our model’s predicted risk of progression, we were able to stratify patients in the validation cohort into high and low risk of progression in the external validation cohort using cluster proportions alone (*P* < 0.001, Fig. 6E; Supplementary Tables S5 and S6). This indicates the applicability of our analysis across multiple treatments, including anti-CD38, which is now standard of care. In addition, in the validation dataset, complete responders (*n* = 8) had a lower predicted risk of progression than patients with very good PR (*n* = 9) or PR (*n* = 3; *P* = 0.045; Fig. 6F). Furthermore, these model hazard scores were significantly lower in patients with sustained MRD negativity (*n* = 10) than in those without sustained MRD negativity (*n* = 10; *P* = 0.002; Fig. 6G). Taken together, cell type proportions in the myeloma microenvironment and their interactions are prognostic in patients following initial therapeutic intervention.

Initial response after ASCT was not sufficient alone to predict the duration of response because, in our dataset in the nonprogressor group, 43% of patients (9/21) were MRD-negative after ASCT and 42% of progressors (8/19) were MRD-negative. The model we have developed is better at predicting progressors based on the tumor microenvironment and may reflect sustained MRD negativity over 12 months (18).

## Discussion

In this study, we have comprehensively described the immune microenvironment in the bone marrow of patients with multiple myeloma undergoing ASCT using single-cell technologies. We analyzed samples both before and after the transplant procedure to detect differences in cell proportions associated with progression as well as correlations between cell proportions and time to progression. These results highlight the importance of increased T-cell populations and decreased monocyte populations, as well as increased T-cell clonotype expansion in patients who progress early.

It is clear that the microenvironment of the bone marrow is increasingly compromised during myeloma progression. Other studies have indicated a change in the composition of the immune cells as the disease progresses from monoclonal gammopathy of undetermined signficance (MGUS) to smoldering multiple myeloma (SMM) to multiple myeloma, although the exact changes differ between studies. Two studies have shown that at the early MGUS and SMM stages, NK cells are increased compared with healthy donors and granzyme B^+^ cytotoxic T cells are decreased (19, 20). However, in high-risk SMM, there is an increase in CD56^dim^ NK cells, CD4^+^ memory and naïve T cells, and CD8^+^ effector memory T cells. Conversely, plasmacytoid dendritic cells, CD14^+^ monocytes, granzyme K^+^ CD8^+^ effector memory cells, and TCR diversity were decreased in high-risk SMM. Another study observed a decrease in memory and naïve CD4^+^ T cells, and an increase in CD8^+^ effector T cells and T-regulatory cells, as well as an enrichment of memory B cells and CD14^+^ and CD16^+^ monocytes as the disease stages progress (21).

A recent study examining the immune microenvironment in multiple myeloma, following treatment with CD38-targeted immunotherapy and proteasome/IMiD combination, showed that the expansion of monocytes, NK-cell activation, and depletion of T cells were associated with sustained MRD negativity (13).

Taken together, these studies could be categorized into high- and low-risk comparisons, whether that is comparing healthy donors and high-risk SMM, a low-risk disease against advanced disease stages, or those with sustained MRD against a lack of MRD negativity. Our study described here can equally be categorized into low-risk nonprogressors versus high risk of progression after ASCT, in which the high-risk group is characterized by increased T-cell infiltration, decreased TCR diversity, decreased monocytes, decreased plasmacytoid dendritic cells, and increased CD56^dim^ NK cell populations. Each of the studies points toward the “high-risk” microenvironment group consisting of this composition of cells, but presumably, the scale of change is different depending on the comparison.

Despite these similarities between datasets, there remains a lot to be learned about the interplay between the immune cell types and the tumor cells themselves. Clearly, the characteristics of the tumor play an important role in disease progression (1), and the interplay between the tumor and the microenvironment can drive both sides (13); determining the role of these interactions will be important going forward. In addition, the microenvironment cells can interact with one another, and in this study, we determine that a model for the combined effect of CD8^+^ exhausted T cells and stromal cells results in the progression of disease by supporting the tumor. Disrupting these T-cell interactions could be a key strategy in targeting the favorable tumor microenvironment, destabilizing the tumor, and making existing therapies more efficacious. Conversely, the relationship between NK cells and pDCs is negatively associated with progression, so promoting these interactions would also be beneficial.

These complex relationships in the microenvironment ultimately influence how multiple myeloma cells can grow and proliferate as they do not exist devoid of the influence of other cell types. Increasingly, it is clear that the microenvironment is essential to multiple myeloma cell growth (5) and evasion of therapy (22). As new immunotherapies, bispecific antibodies, and chimeric antigen receptor and TCR therapies become more widely available, it will become more important to understand which native immune cell types in the microenvironment are regulating growth and resistance and may affect these new treatments. In this study, we shed new light and describe in detail the complex interplay among monocytes, T-cell subtypes, and NK cells that can affect progression after ASCT.

The model described here was generated through machine learning and validated in an additional independent dataset, showing the importance of analyzing data in such a way that identifies relationships that may be difficult to determine otherwise. Although we devised a model of progression based on scRNA and TCR sequencing data, the same rationale could be applied to existing flow cytometry data using the EuroFlow MRD panel. We have shown that this existing assay, which is performed routinely in many centers across the world, correlates well with scRNA sequencing data and could be used as a biomarker for the risk of progression.

Another area of interest will be whether the increased number of T-cell subsets that are associated with progression can be utilized to a therapeutic advantage. Perhaps the increased T-cell infiltration can be used in conjunction with T cell–engaging treatments, such as bispecific antibodies, to effectively control the disease in patients who are likely to progress quickly after ASCT.

## Supplementary Material

Supplementary Figure S1 Supplementary Figure S1

Supplementary Figure S2 Supplementary Figure S2

Supplementary Figure S3 Supplementary Figure S3

Supplementary Figure S4 Supplementary Figure S4

Supplementary Figure S5 Supplementary Figure S5

Supplementary Table S1 Supplementary Table S1

Supplementary Table S2 Supplementary Table S2

Supplementary Table S3 Supplementary Table S3

Supplementary Table S4 Supplementary Table S4

Supplementary Table S5 Supplementary Table S5

Supplementary Table S6 Supplementary Table S6
