# Supplementary material for: Identification of the Distinct Immune Microenvironment Features Associated with Progression Following High-Dose Melphalan and Autologous Stem Cell Transplant in Multiple Myeloma
Source: Cancer Immunol Res. 2025 May 8;13(7):1070–9. doi: 10.1158/2326-6066.CIR-25-0019 (PMC12214876; doi:10.1158/2326-6066.CIR-25-0019)

Supplementary Figure S1. A. Days between pre-transplant to post-transplant sample collection for each patient. The day of transplant is shown as 0. Samples are colored based on progression status **B.** UMAP of CD138- BME cells colored by major cell types. **C.** A dotplot of the mean expression of highly represented individual marker genes per cluster. **D.** Percentage of cells per cluster in pre- vs. post-ASCT samples. **E.** Major cell clusters with significant mean percentage difference between Pre-ASCT and Post-ASCT samples.

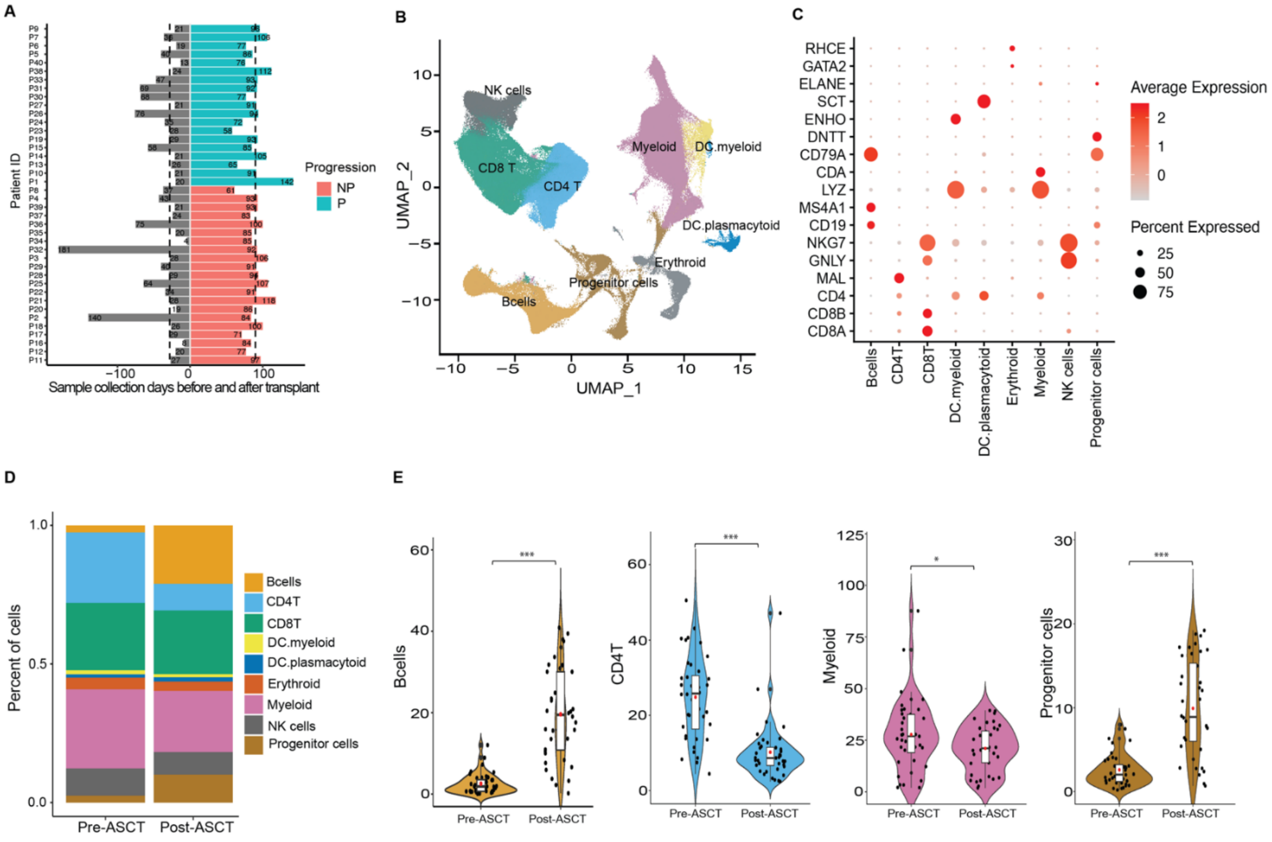

Supplement: Supplementary Figure S1 [file cir-25-0019_supplementary_figure_s1_supps1.pdf]
