# Supplementary material for: Identification of the Distinct Immune Microenvironment Features Associated with Progression Following High-Dose Melphalan and Autologous Stem Cell Transplant in Multiple Myeloma
Source: Cancer Immunol Res. 2025 May 8;13(7):1070–9. doi: 10.1158/2326-6066.CIR-25-0019 (PMC12214876; doi:10.1158/2326-6066.CIR-25-0019)

Supplementary Figure S2. Flow cytometry gating strategy for MRDflow. A. Initially, all plasma cells are captured based on SSC, FSC, and expression of CD38, CD138 and CD45 antigens (shown as gates in yellow). These events are designated as total normal plasma cells. B. Subsequently, focusing on plasma cell population only, the operator looks for plasma cells with abnormal antigen expression taking into account CD19, CD56, CD117, kappa and lambda antigens (normal plasma cells shown in pink, neoplastic plasma cells shown in yellow). In order to reduce debris, the SSC/FSC scattergram is used to gate in nucleated cells and gate out debris, and FSC peak vs. FSC area scattergram to remove doublets.

A

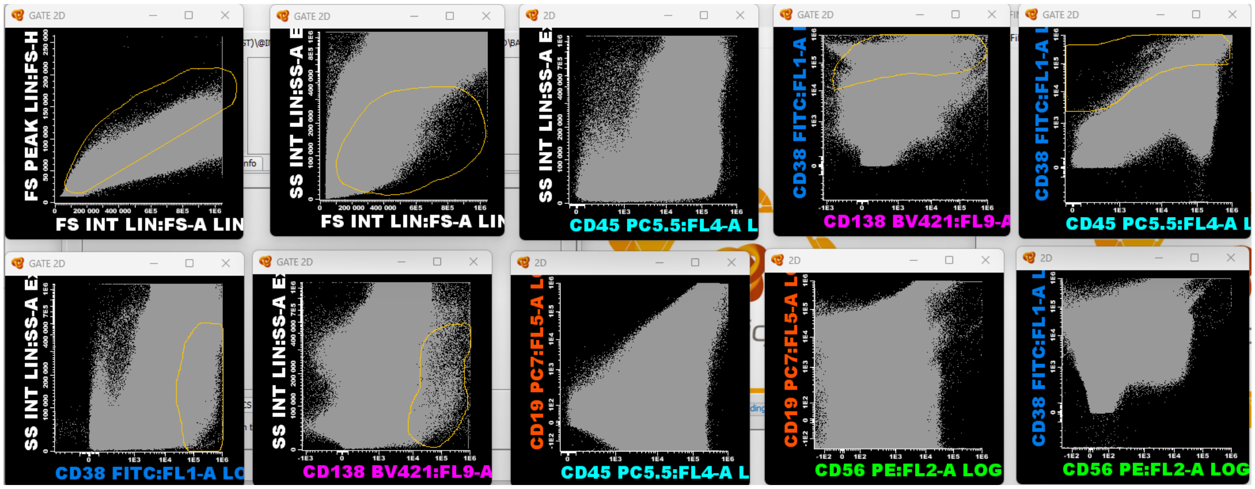

B

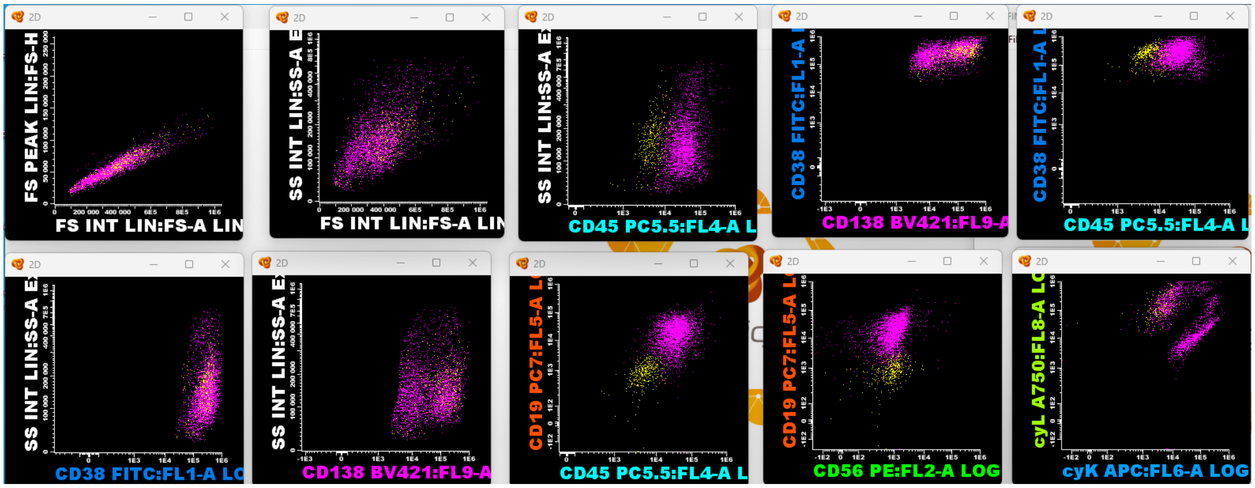

Supplement: Supplementary Figure S2 [file cir-25-0019_supplementary_figure_s2_supps2.pdf]
