# Supplementary material for: Identification of the Distinct Immune Microenvironment Features Associated with Progression Following High-Dose Melphalan and Autologous Stem Cell Transplant in Multiple Myeloma
Source: Cancer Immunol Res. 2025 May 8;13(7):1070–9. doi: 10.1158/2326-6066.CIR-25-0019 (PMC12214876; doi:10.1158/2326-6066.CIR-25-0019)

Supplementary Figure S3. Identifying sub-clusters of cell types associated with progression. A. Identification and mapping of cell type sub-clusters. B. Mean proportions of cells and in progressor (P) and non-progressor (NP) samples at both pre- and post-ASCT timepoints. # = any significant difference. C. Cell types with significant differences in cell proportions between response groups at each timepoint. *=P<0.05, **=P<0.01, ***=P<0.001. Cell proportions were calculated per sample based on the number of cells in that cluster divided by the total number of cells in that sample.

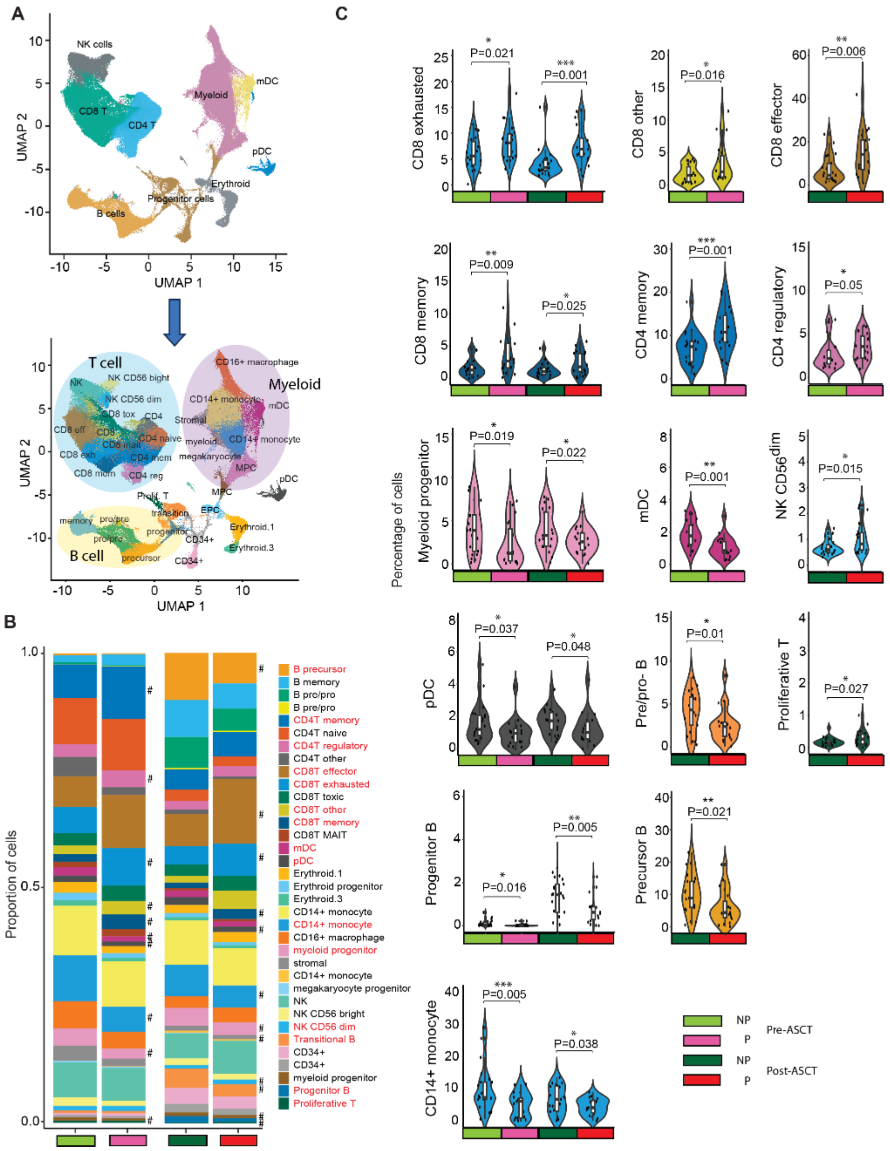

Supplement: Supplementary Figure S3 [file cir-25-0019_supplementary_figure_s3_supps3.pdf]
