# Supplementary figures and images for: Identification of the Distinct Immune Microenvironment Features Associated with Progression Following High-Dose Melphalan and Autologous Stem Cell Transplant in Multiple Myeloma
Source: Cancer Immunol Res. 2025 May 8;13(7):1070–9. doi: 10.1158/2326-6066.CIR-25-0019 (PMC12214876; doi:10.1158/2326-6066.CIR-25-0019)

**Supplementary Figure S4. T cell annotation using marker genes.**

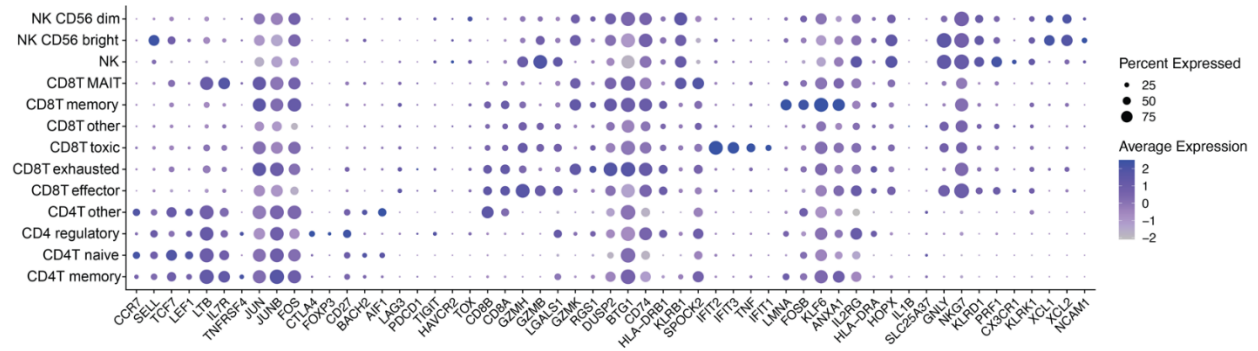

Supplement: Supplementary Figure S4 [file cir-25-0019_supplementary_figure_s4_supps4.pdf]
