# Supplementary material for: Identification of the Distinct Immune Microenvironment Features Associated with Progression Following High-Dose Melphalan and Autologous Stem Cell Transplant in Multiple Myeloma
Source: Cancer Immunol Res. 2025 May 8;13(7):1070–9. doi: 10.1158/2326-6066.CIR-25-0019 (PMC12214876; doi:10.1158/2326-6066.CIR-25-0019)

Supplementary Figure S5. Progression is associated with increased proportions of CD8+ T cells. A. T cell clusters and cell types. B. T cells annotated by TCR sequence to identify single and expanded clonotypes. C. Count of unique clonotypes per sample in each group. D. Chao1 diversity index per sample in each group.

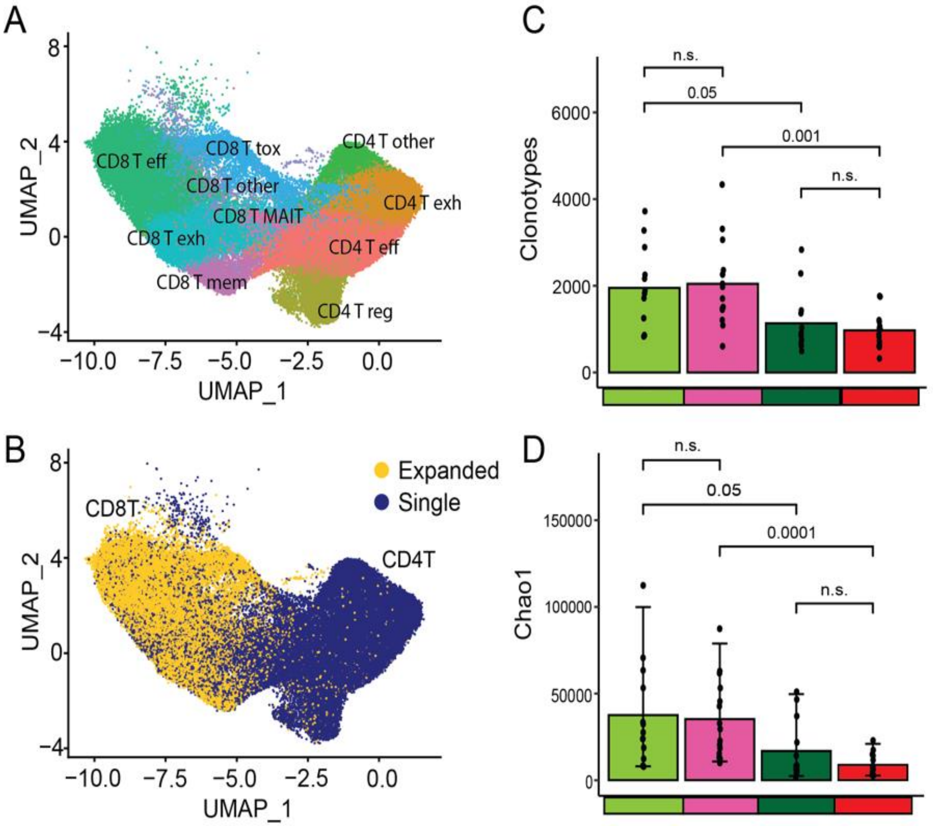

Supplement: Supplementary Figure S5 [file cir-25-0019_supplementary_figure_s5_supps5.pdf]
