# Supplementary material for: Identification of the Distinct Immune Microenvironment Features Associated with Progression Following High-Dose Melphalan and Autologous Stem Cell Transplant in Multiple Myeloma
Source: Cancer Immunol Res. 2025 May 8;13(7):1070–9. doi: 10.1158/2326-6066.CIR-25-0019 (PMC12214876; doi:10.1158/2326-6066.CIR-25-0019)
Supplement: Supplementary Table S2 [file cir-25-0019_supplementary_table_s2_suppst2.pdf]

Supplementary Table S2: Cell type mapping between datasets due to inexact correspondence.

Sudha et al.	Maura et al.
NK.CD56.bright	CD56.bright.NK
NK	NK
CD4 T regulatory	Treg
CD8 T effector	CD8.Effector_1<br>CD8.Effector_2
CD8 T exhausted	CD8.Memory_1[1]
mDC	cDC2
CD14+ monocyte	CD14.Mono
CD8T other	gdT[2]
CD16+ monocyte	CD16.Mono
Stromal	LMPP[3]
CD14+ monocyte	CD14.Mono
pDC	pDC
CD4T memory	CD4.Memory
Proliferative T	HSC[4]
CD8T memory	CD8.Memory_1<br>CD8.Memory_2

[1]https://doi.org/10.1038/s41577-019-0221-9 **Similarities between memory T cells and exhausted T-cell precursors. Note that there are no exhausted T-cell clusters annotated in this dataset.**

[2]**gdT was selected as the closest approximation to the T-cell other class in our dataset**

[3]**Lmypho-myeloid primed progenitor (LMPP) was selected as the closest approximation to the Myeloid stromal class**

[4]**The closest approximation to our proliferative T-cell cluster was the stem cell cluster.**
