# Supplementary material for: Identification of the Distinct Immune Microenvironment Features Associated with Progression Following High-Dose Melphalan and Autologous Stem Cell Transplant in Multiple Myeloma
Source: Cancer Immunol Res. 2025 May 8;13(7):1070–9. doi: 10.1158/2326-6066.CIR-25-0019 (PMC12214876; doi:10.1158/2326-6066.CIR-25-0019)
Supplement: Supplementary Table S3 [file cir-25-0019_supplementary_table_s3_suppst3.pdf]

Supplementary Table S3. Major cell population proportions in pre- and post-ASCT samples.

	Pre-ASCT						Post-ASCT				
**Cell type**	Cluster	Median (P)	Median (NP)	P vs NP	Adjusted P-value (FDR <0.25)	q value	Median (P)	Median (NP)	P vs NP	Adjusted P-value (FDR <0.25)	q value
**B cells**	B cells	1.865	1.893	n.s.	n.s.	0.108	15.790	21.388	0.014	0.063	0.066
**CD4 T cells**	CD4 T	29.686	20.024	0.026	0.058	0.072	9.350	7.195	n.s.	n.s.	0.122
**CD8 T cells**	CD8 T	33.653	18.838	0.004	0.018	0.072	28.873	12.660	0.004	0.036	0.066
**Myeloid dendritic cells**	mDC	0.847	1.762	0.001	0.009	0.072	1.035	1.359	n.s.	n.s.	0.066
**Plasmacytoid dendritic cells**	pDC	0.995	1.201	0.037	0.066	0.072	1.009	1.735	0.048	0.108	0.066
**Erythroid cells**	Erythroid	2.584	3.621	n.s.	n.s.	0.097	2.043	3.253	n.s.	n.s.	0.122
**Myeloid cells**	Myeloid	21.720	27.880	0.009	0.026	0.072	20.022	24.102	n.s.	n.s.	0.066
**NK cells**	NK cell	7.730	7.781	n.s.	n.s.	0.108	6.897	7.912	n.s.	n.s.	0.069
**Progenitor cells**	Progenitor	1.627	2.824	n.s.	n.s.	0.097	7.262	12.866	0.022	0.066	0.066
