# Supplementary material for: Identification of the Distinct Immune Microenvironment Features Associated with Progression Following High-Dose Melphalan and Autologous Stem Cell Transplant in Multiple Myeloma
Source: Cancer Immunol Res. 2025 May 8;13(7):1070–9. doi: 10.1158/2326-6066.CIR-25-0019 (PMC12214876; doi:10.1158/2326-6066.CIR-25-0019)
Supplement: Supplementary Table S4 [file cir-25-0019_supplementary_table_s4_suppst4.pdf]

Supplementary Table S4. Cell sub-population proportions in pre- and post-ASCT samples with significant differences.

		Pre-ASCT					Post-ASCT				
**Cell type**	Cluster	Median (P)	Median (NP)	P vs NP	Adjuste P-value (FDR <0.25)	q value	Median (P)	Median (NP)	P vs NP	Adjusted P-value (FDR <0.25)	q value
**Precursor B**	Bcell.1	0.185	0.454	n.s.	n.s.	0.069	6.112	10.278	0.013	0.087	0.052
Memory B	Bcell.2	2.292	1.606	n.s.	n.s.	0.073	4.757	6.367	n.s.	n.s.	0.072
Transitional B	Bcell.3	0.167	0.414	n.s.	n.s.	0.069	4.469	6.315	n.s.	n.s.	0.053
Transitional B	Bcell.4	0.025	0.025	n.s.	n.s.	0.149	0.244	0.306	n.s.	n.s.	0.072
CD4 T mem	CD4T.1	11.415	7.018	0.001	0.017	0.053	5.143	4.469	n.s.	n.s.	0.083
CD4 T naive	CD4T.2	11.624	9.124	n.s.	n.s.	0.069	2.094	2.993	n.s.	n.s.	0.089
CD4 T reg	CD4T.3	3.512	2.667	0.05	0.185	0.066	2.232	2.147	n.s.	n.s.	0.136
CD4 T others	CD4T.4	1.781	2.855	n.s.	n.s.	0.072	0.466	0.816	n.s.	n.s.	0.060
CD8 T eff	CD8T.1	10.255	7.033	n.s.	n.s.	0.069	14.433	7.296	0.006	0.069	0.052
CD8T exh	CD8T.2	8.296	6.057	0.021	0.091	0.053	7.552	4.241	0.001	0.035	0.052
CD8 Tox	CD8T.3	3.247	2.191	n.s.	n.s.	0.069	3.108	2.318	n.s.	n.s.	0.089
CD8 T other	CD8T.4	3.443	1.723	0.016	0.091	0.053	4.512	1.814	n.s.	n.s.	0.053
CD8 T mem	CD8T.5	3.465	1.636	0.009	0.078	0.053	2.277	1.330	0.025	0.105	0.052
CD8 MAIT	CD8T.6	1.487	1.244	n.s.	n.s.	0.089	0.444	0.447	n.s.	n.s.	0.150
Myeloid DC	mDC	1.063	1.868	0.001	0.017	0.053	1.091	1.382	n.s.	n.s.	0.061
Plasmacytoid DC	pDC	0.982	1.587	0.037	0.143	0.060	1.240	1.719	0.048	0.152	0.053
	Erythroid.1	2.113	2.549	n.s.	n.s.	0.115	2.311	1.764	n.s.	n.s.	0.099
Erythroid progenitor	Erythroid.2	1.082	1.487	n.s.	n.s.	0.073	0.669	0.916	n.s.	n.s.	0.080
	Erythroid.3	0.847	1.243	n.s.	n.s.	0.077	0.636	0.704	n.s.	n.s.	0.120
CD14+ mono	Myeloid.1	9.348	11.407	n.s.	n.s.	0.072	7.384	8.840	n.s.	n.s.	0.078
CD14+ mono	Myeloid.2	4.859	9.887	0.005	0.058	0.053	4.458	6.458	0.038	0.133	0.053
CD16+ / macrophage	Myeloid.3	2.923	5.456	n.s.	n.s.	0.069	3.022	2.489	n.s.	n.s.	0.089
Myeloid Progenitor	Myeloid.4	2.236	4.067	0.019	0.091	0.053	2.637	3.897	0.022	0.105	0.052
Stromal	Myeloid.5	1.415	2.622	n.s.	n.s.	0.072	0.856	0.943	n.s.	n.s.	0.105
CD14+ monocyte	Myeloid.6	0.082	0.085	n.s.	n.s.	0.147	0.197	0.369	0.05	0.157	0.053
Megak.Prog -> Platelet	Myeloid.7	0.206	0.317	n.s.	n.s.	0.069	0.118	0.140	n.s.	n.s.	0.113
NK	NK.1	7.142	7.801	n.s.	n.s.	0.115	7.125	5.511	n.s.	n.s.	0.072
NK.CD56.bright	NK.2	1.133	1.684	n.s.	n.s.	0.072	1.302	1.430	n.s.	n.s.	0.113
NK.CD56.dim	NK.3	1.108	1.000	n.s.	n.s.	0.108	1.052	0.695	0.015	0.087	0.052
Transitional B	Prog.1	0.408	0.556	n.s.	n.s.	0.073	2.551	4.164	0.011	0.087	0.052
Progenitor (CD34+)	Prog.2	0.448	0.563	n.s.	n.s.	0.108	2.504	3.334	n.s.	n.s.	0.059
Progenitor (CD34+)	Prog.3	0.401	0.504	n.s.	n.s.	0.108	1.227	1.790	n.s.	n.s.	0.054
Myeloid Progenitor	Prog.4.GMP	0.590	0.787	n.s.	n.s.	0.072	0.740	0.802	n.s.	n.s.	0.120
Progenitor B	Prog.5	0.036	0.122	0.016	0.091	0.053	0.722	1.332	0.005	0.069	0.052
Proliferative T	Prog.6	0.386	0.360	n.s.	n.s.	0.142	0.314	0.185	0.027	0.105	0.052
