# Supplementary material for: Identification of the Distinct Immune Microenvironment Features Associated with Progression Following High-Dose Melphalan and Autologous Stem Cell Transplant in Multiple Myeloma
Source: Cancer Immunol Res. 2025 May 8;13(7):1070–9. doi: 10.1158/2326-6066.CIR-25-0019 (PMC12214876; doi:10.1158/2326-6066.CIR-25-0019)
Supplement: Supplementary Table S5 [file cir-25-0019_supplementary_table_s5_suppst5.pdf]

Supplementary Table S5: Log rank test results by cutoff

Percentile cutoff	P
0.50	1.69E-1
0.55	1.21E-1
0.60	7.88E-2
0.65	4.50E-2
0.70	2.06E-2
0.75	6.22E-3
0.80	7.64E-4
0.85	6.31E-6
0.90	6.33E-5
0.95	6.33E-5
