# Supplementary material for: Identification of the Distinct Immune Microenvironment Features Associated with Progression Following High-Dose Melphalan and Autologous Stem Cell Transplant in Multiple Myeloma
Source: Cancer Immunol Res. 2025 May 8;13(7):1070–9. doi: 10.1158/2326-6066.CIR-25-0019 (PMC12214876; doi:10.1158/2326-6066.CIR-25-0019)
Supplement: Supplementary Table S6 [file cir-25-0019_supplementary_table_s6_suppst6.pdf]

Supplementary Table S6: T-cell clone proportion

Samples	Batch	Disease progression	Clones	Percentage	Clonal.count.prop
0661-206	Pre-ASCT	DP	1	10.7	0.000689655
0661-206	Post-ASCT	DP	2	10	0.00113443
0661-368	Pre-ASCT	DP	1	10.7	0.000586167
0661-368	Post-ASCT	DP	1	21.7	0.001015228
0661-463	Pre-ASCT	NP	5	10.3	0.001730104
0661-463	Post-ASCT	NP	4	11.1	0.004728132
0661-532	Pre-ASCT	NP	1	18	0.000528262
0661-532	Post-ASCT	NP	1	27.1	0.001160093
0661-589	Pre-ASCT	NP	11	10.1	0.012761021
0661-589	Post-ASCT	NP	34	10.1	0.037321625
0661-593	Pre-ASCT	NP	2	11.5	0.002347418
0661-593	Post-ASCT	NP	3	11.3	0.004792332
0661-620	Pre-ASCT	DP	9	10.4	0.003984064
0661-620	Post-ASCT	DP	4	12	0.002284409
0661-621	Pre-ASCT	DP	5	10.5	0.002117747
0661-621	Post-ASCT	DP	2	11.4	0.001657001
0661-627	Pre-ASCT	DP	102	10	0.069199457
0661-627	Post-ASCT	DP	2	10.6	0.003278689
0661-747	Pre-ASCT	NP	89	10.1	0.041108545
0661-747	Post-ASCT	NP	9	10.3	0.011952191
0661-750	Pre-ASCT	DP	1	16	0.000301841
0661-750	Post-ASCT	DP	1	11.1	0.001375516
0661-753	Pre-ASCT	DP	69	10	0.015909615
0661-753	Post-ASCT	DP	15	10.1	0.014018692
0661-757	Pre-ASCT	NP	210	10	0.056436442
0661-757	Post-ASCT	NP	98	10	0.042888403
0661-878	Pre-ASCT	DP	4	10.1	0.002649007
0661-878	Post-ASCT	DP	9	10.2	0.010238908
0661-900	Pre-ASCT	DP	3	11.6	0.001323919
0661-900	Post-ASCT	DP	1	11.7	0.001212121
0661-903	Pre-ASCT	NP	4	10.9	0.004801921
0661-903	Post-ASCT	NP	6	11.5	0.009693053
0661-1050	Pre-ASCT	DP	41	10.1	0.037614679
0661-1050	Post-ASCT	DP	4	11.2	0.012345679
0661-1063	Pre-ASCT	DP	5	10.9	0.002526529
0661-1063	Post-ASCT	DP	2	11.5	0.002002002
0661-1065	Pre-ASCT	NP	6	10.6	0.003278689
0661-1065	Post-ASCT	NP	4	10.6	0.005856515
0661-1068	Pre-ASCT	NP	28	10.2	0.015334064
0661-1068	Post-ASCT	NP	14	10.2	0.010248902

0661-1084	Pre-ASCT	NP	80	10.1	0.024405125
0661-1084	Post-ASCT	NP	61	10	0.059223301
0661-1094	Pre-ASCT	DP	82	10	0.040058622
0661-1094	Post-ASCT	DP	4	11	0.003415884
0661-1102	Pre-ASCT	DP	5	11.4	0.004132231
0661-1102	Post-ASCT	DP	3	13.3	0.00456621
0661-1121	Pre-ASCT	NP	11	10.1	0.004882379
0661-1121	Post-ASCT	NP	1	12.2	0.000698812
0661-1129	Pre-ASCT	DP	2	15.1	0.003305785
0661-1129	Post-ASCT	DP	1	18.4	0.001703578
0661-1154	Pre-ASCT	DP	84	10	0.027424094
0661-1154	Post-ASCT	DP	4	10.7	0.003910068
0661-1162	Pre-ASCT	NP	9	10.3	0.007177034
0661-1162	Post-ASCT	NP	5	11.1	0.010060362
0661-1163	Pre-ASCT	NP	15	10.3	0.008741259
0661-1163	Post-ASCT	NP	11	10.2	0.00388144
